# Traumatic Atlantoaxial Dislocation with Type II Odontoid Fractures: A Case Report

**DOI:** 10.4314/ejhs.v30i6.25

**Published:** 2020-11

**Authors:** Aldhi Tri Budhi, Nasrullah, Andi Asadul Islam, Djoko Widodo, Willy Adhimarta, Andi Ihwan, Muhammad Faruk

**Affiliations:** 1 Department of Surgery, Faculty of Medicine, Hasanuddin University, Makassar, Indonesia; 2 Division of Neurosurgery, Department of Surgery, Faculty of Medicine, Hasanuddin University, Makassar, Indonesia

**Keywords:** Cervical spine injury, Odontoid fracture, Hemiparesis

## Abstract

**Background:**

Odontoid fracture frequently ensues after a cervical trauma, and most commonly at the junction between the dens and the body (type II odontoid fracture).

**Case Presentation:**

This report is focused on a 24-years-old male patient with right-sided hemiparesis, resulting from traumatic atlantoaxial dislocation with type II odontoid fracture. Cervical CT-scanning showed a spondylolisthesis of the C1-C2 complex with type II odontoid fracture, and the injury was treated using posterior reduction and internal stabilization. Therefore, hemiparesis was reduced, and during the follow-up period, our patients were disease-free.

**Conclusion:**

Early diagnosis and the appropriate management of atlantoaxial trauma is a possible approach towards preventing severe neurological deficits.

## Introduction

Odontoid fractures occur frequently after cervical trauma, and the most common type is reported at the junction between the dens and the body (type II odontoid fracture) (1). This phenomenon is possibly associated with spondylolisthesis or retrolisthesis of the C1-C2 complex, in relation to the C2 body. In addition, the fracture has been affiliated with the incidence of spinal cord compression, which leads to severe neurological deficits (2). Here, we report the uncommon case of a young male patient suffering right-sided hemiparesis, after a traumatic atlantoaxial dislocation accompanied by type II odontoid fracture.

## Case Report

**Figure 1:**
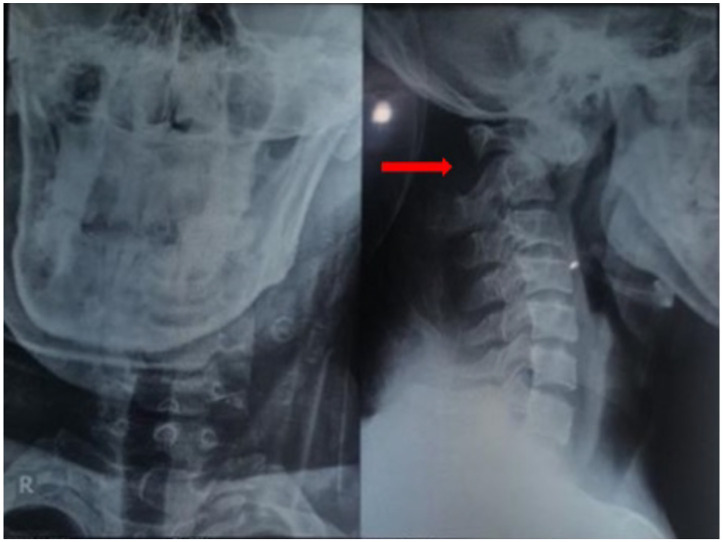
A/P Lateral X-rays of the cervical spine showed fracture at the axis (C2), specifically at the odontoid process, categorized as type II, based on Anderson and D'Alonzo classification (arrows).

**Figure 2:**
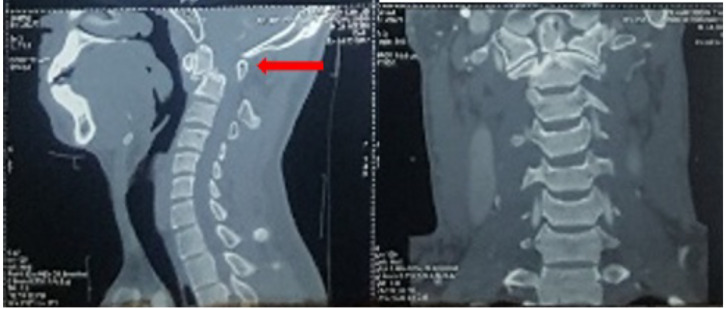
Sagittal-coronal planes CT scan of the cervical spine showed fracture at the axis (C2), specifically at the odontoid process, categorized as type II, based on Anderson and D'Alonzo classification (arrows).

A 24 years old male patient was conveyed by an ambulance to the hospital after receiving a blunt trauma at the posterior of the neck, following a fall accident. The examination on arrival at the emergency department showed normal blood pressure and a heart rate of 92 beats per minute (bpm), and also a Glasgow Coma Score of 15, with right-sided hemiparesis. In addition, the cervical spine X-rays (Figure 1) and CT-scan (Figure 2) showed fracture at the axis (C2), specifically at the odontoid process, categorized as type II, based on the Anderson and D'Alonzo classification.

This was a conservative treatment to reduce the atlantoaxial dislocation, involving axial traction alongside a halo head brace. Also, the joint showed no signs of reduction, despite the progressive increase in traction weight by up to 4 kg for seven days.

**Figure 3:**
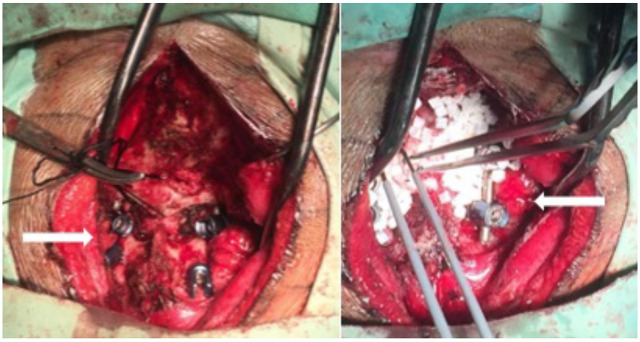
Posterior pedicle screw fixation of C1-C2 with bone graft

Following the traction failure, surgery was opted to decrease the dislocation and repair the C1-C2 joint, before placing the patient in a prone position, to perform the posterior approach. This process was completed by fixing four pedicle screws into the joint line articular processes, followed by bone graft fusion to the space (Figure 3).

**Figure 4:**
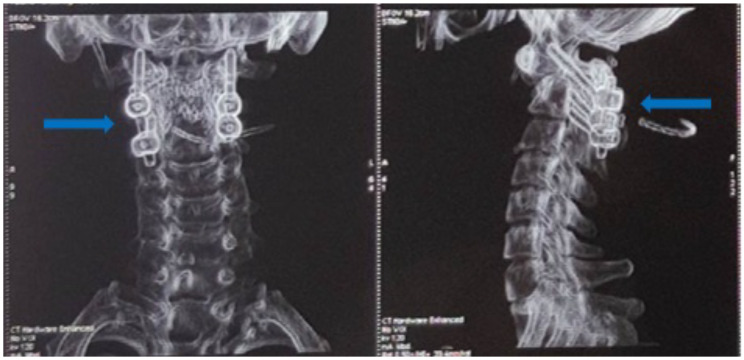
CT-scan postoperative of the cervical spine coronal-sagittal showed the reduction of C1-C2 joint.

A control CT-scan was performed one-week post-operative (Figure 4), confirming the reduction of C1-C2 joint. Also, the odontoid process was verified to have properly aligned with the C2 vertebral body. In addition, immobilization necessitating a flexible Minerva brace was used to moderate the pain for six weeks, alongside hemiparesis reduction, and the patient was subsequently discharge three weeks post-surgery.

Follow-up up to 12 months after surgery, based on clinical evaluation, the patient was stable, as the X-rays did not recognize any fixation failures or secondary displacement. Despite the stiff fixation system, the patient experienced no major functional challenges, and the dynamic X-ray showed adequate fixation. Our patients were disease-free, had full range of motion, and no neck pain felt.

## Discussion

An odontoid fracture occurs frequently as a result of cervical trauma, and the most common type ensues at the junction between the dens and the body (type II odontoid fracture) (1). Type II odontoid fractures are the most usual type of odontoid fractures in the older patients (more than 60 years of age) (2).

The cervical spine X-rays of this case report showed fracture at the odontoid process, and was categorized as type II, based on the Anderson and D'Alonzo classification. In addition, the atlas (C1) demonstrated an anterior displacement in relation to the C2, while the fracture was linked to the Type IV rotational atlantoaxial dislocation. These evaluations were based on Fielding classification (3).

An odontoid fracture is possibly correlated with spondylolisthesis or retrolisthesis of the C1-C2 complex, in relation to the C2 body, resulting in spinal cord compression, and also the production of severe neurological deficits. In addition, 82% of patients with type II fractures present with intact neurological status, and 8% demonstrate minimal sensory disturbances over the scalp or limb, while 10% experience significant neurological deficits (4).

This instigates the selection of closed reduction technique, where a halo head brace is used to provide traction, with a steadily increasing weight, up to 10% of the total bodyweight. Fielding and Hawkins (3,5) recommended < 9 kg for adults, and a successful procedure is progressed with a focus on the C2 fracture (2). However, a failed therapy, featuring poor displacement possibly requires additional conservative treatments with a Minerva brace for three months, which prompts quick reduction of C1-C2 dislocation. This management approach provides enough time for the complete healing of cervical spine ligaments. Also, surgical anterior screw fixation of the odontoid is needed after the detection of odontoid fracture displacement of instability, using the French anagram for Oblique Below and Backward [OBAR] or horizontal fracture [HTAL] type fracture. This technique has not previously been reported in combination with C1-C2 dislocation (1,2).

A posterior approach like in our case is recommended to stabilize odontoid fractures classified to be non-amenable through anterior screw fixation. This commonly involves creating a bone graft between the C1-C2 lamina posterior arch, using sublaminar wiring (2,4). However, the posterior approach possesses numerous advantages, including the permission of access to the posterior joint affected by a dislocation, and also the clearance for C1-C2 screw fixation. The posterior cervical stabilization procedure provides a 77 to 90% fusion rate in type II odontoid fracture (2).

The injury combination of type II odontoid process fracture and atlantoaxial joint dislocation is uncommon. However, treatment involves conducting a closed reduction on the dislocation, and a successful remediation is followed by therapy for the odontoid fracture. The treatment was continued with conservative management or direct screw fixation, after accessing the extent of reduction and joint stability. However, failure requires the conduction of open reduction through a posterior approach. In addition, early diagnosis and appropriate atlantoaxial trauma management have been implicated in the prevention of more severe neurological deficits.
